# Oral and Parenteral vs. Parenteral Antibiotic Prophylaxis for Patients Undergoing Laparoscopic Colorectal Resection: An Intervention Review with Meta-Analysis

**DOI:** 10.3390/antibiotics11010021

**Published:** 2021-12-24

**Authors:** Giuseppe Sangiorgio, Marco Vacante, Francesco Basile, Antonio Biondi

**Affiliations:** Department of General Surgery and Medical-Surgical Specialties, University of Catania, Via S. Sofia 78, 95123 Catania, Italy; marcovacante@yahoo.it (M.V.); fbasile@unict.it (F.B.); abiondi@unict.it (A.B.)

**Keywords:** laparoscopy, colorectal neoplasm resection, antibiotic prophylaxis, surgical wound infection, postoperative complications

## Abstract

This study aims to systematically assess the efficacy of parenteral and oral antibiotic prophylaxis compared to parenteral-only prophylaxis for the prevention of surgical site infection (SSI) in patients undergoing laparoscopic surgery for colorectal cancer resection. Published and unpublished randomized clinical trials comparing the use of oral and parenteral prophylactic antibiotics vs. parenteral-only antibiotics in patients undergoing laparoscopic colorectal surgery were collected searching electronic databases (MEDLINE, CENTRAL, EMBASE, SCIENCE CITATION INDEX EXPANDED) without limits of date, language, or any other search filter. The outcomes included SSIs and other infectious and noninfectious postoperative complications. Risk of bias was assessed using the Cochrane revised tool for assessing risk of bias in randomized trials (RoB 2). A total of six studies involving 2252 patients were finally included, with 1126 cases in the oral and parenteral group and 1126 cases in the parenteral-only group. Meta-analysis results showed a statistically significant reduction of SSIs (OR 0.54, 95% CI 0.40 to 0.72; *p* < 0.0001) and anastomotic leakage (OR 0.55, 95% CI 0.33 to 0.91; *p* = 0.02) in the group of patients receiving oral antibiotics in addition to intravenous (IV) antibiotics compared to IV alone. Our meta-analysis shows that a combination of oral antibiotics and intravenous antibiotics significantly lowers the incidence of SSI compared with intravenous antibiotics alone.

## 1. Introduction

Surgical site infection (SSI) is defined as an infection of superficial or deep tissue or organs at the surgical site or related to the site of the surgical procedure [1]. SSI is quite common in people undergoing colorectal surgery and is related to increased length of stay and costs and poor quality of life [2,3,4]. Risk factors for wound infection include male sex, advanced age, previous chemotherapy, conversion from laparoscopic to open technique, and reintervention within the first 30 postoperative days [2,3,4]. Antibiotic prophylaxis has been shown to have a pivotal role for preventing SSIs in patients undergoing elective colorectal surgery [5,6,7]. In several studies, authors reported evidence regarding the effect of oral and parenteral prophylaxis in colorectal surgery [8,9,10], but none of them referred to laparoscopic surgery. In fact, laparoscopic surgery can achieve similar oncological outcomes as compared with open surgery [11,12] and showed potential advantages, including less bleeding, faster postoperative recovery, less pain, and less wound-related complications [13,14,15,16]. However, further research is needed on the effectiveness of pre-operative oral antibiotic regimen in patients undergoing elective laparoscopic colon surgery.

Another frequent postoperative complication of laparoscopic colon surgery is anastomotic leakage (AL) [17]. AL, which is defined as a defect of the intestinal wall at the anastomotic site, is represented by a communication between the intra- and extraluminal compartments [18]. The rates of AL could vary from 3% to 30% according to the site, technique, and type of surgery [19]. AL is an important cause of postoperative morbidity and mortality, and the occurrence of AL could raise the risk of local recurrence and need for reintervention, therefore having a major impact on the quality of life [20]. Other postoperative complications can be divided into infectious and noninfectious [21]. These may include pneumonia, urinary tract disorders, enteritis/colitis, and bowel obstruction. The main goal of pre- and postoperative care, if properly carried out, is to avoid the onset of such complications [22,23,24]. There is strong evidence that prophylactic antibiotic use is effective in reducing SSI rates, thus leading to reductions in hospitalization time and cost [25,26]. We hypothesized that the administration of oral and intravenous antibiotics vs. intravenous antibiotics alone could be more advantageous in reducing the incidence of SSI and other complications, such as AL, in patients undergoing laparoscopic colorectal surgery.

The aim of the current study was to evaluate the available evidence from randomized controlled trials comparing the effectiveness of combined oral and intravenous antibiotics vs. intravenous antibiotics alone in reducing the incidence of SSI and other complications following laparoscopic colorectal surgery.

## 2. Materials and Methods

The Preferred Reporting Items for Systematic reviews and Meta-Analyses (PRISMA) 2020 guidelines [27] were used to conduct this systematic review.

### 2.1. Eligibility Criteria

Criteria for considering studies for this review were the following:

1. Types of studies
  All randomized clinical trials (RCTs). Nonrandomized and quasirandomized trials were excluded. Studies where it was not possible to obtain the complete manuscript were excluded.
2. Types of participants
  Patients undergoing laparoscopic colorectal surgery.
3. Types of interventions and comparison
  Oral and parenteral antibiotic prophylaxis vs. parenteral-only prophylaxis.
4. Types of outcome measures
  Primary outcomes: SSIs. Secondary outcomes: other infectious and noninfectious postoperative complications. Studies with no outcome of interest were excluded.

### 2.2. Search Strategy

The Peer Review of Electronic Search Strategies (PRESS) Guideline [28] was consulted for the search strategy development process. The electronic databases MEDLINE, The Cochrane Central Register of Controlled Trials (CENTRAL), EMBASE, and Science Citation Index Expanded (SCI-EXPANDED) were searched without limits of date, language, or any other search filter. Searches started on the 1 May 2021 and were updated on 6 December 2021. Searches were performed by two reviewers (G.S. and M.V.) independently. Search strategies with the time span of the searches are showed in Appendix A. Other electronic searches that were performed included trial registers for ongoing and registered trials such as ClinicalTrials.gov (clinicaltrials.gov) and World Health Organization (WHO). Additionally, reference lists of included articles were hand-searched and experts in the field were contacted to obtain additional data.

### 2.3. Selection Process

The review was conducted according to the recommendations of the Cochrane Handbook for Systematic Reviews of Interventions 6.2 [29]. Two reviewers (G.S. and M.V.) independently selected studies according to the inclusion and exclusion criteria. An initial screening of the titles and abstracts was performed. Full texts of all potentially eligible studies were retrieved and examined to decide whether the studies were eligible for inclusion in the review. Disagreements were resolved through discussion or by a third review author (A.B.). No automation tools were used in the process.

### 2.4. Data Collection Process

Two reviewers (G.S. and M.V.) independently extracted the data from eligible studies using a data preformatted form designed by the review authors. Any discrepancies in results were resolved by repeating data extraction and discussion. Data extracted included: characteristics of study (first author, year of publication, study design), number of participants, types of surgical resection, outcome data, and details of antibiotic regimes administered.

### 2.5. Risk of Bias Assessment

Review authors (G.S. and M.V.) independently assessed the included studies for risk of bias using the Cochrane revised tool for assessing risk of bias in randomized trials (RoB 2) [30] to assess five domains:

1. Bias arising from the randomization process;
2. Bias due to deviations from intended interventions;
3. Bias due to missing outcome data;
4. Bias in measurement of the outcome;
5. Bias in selection of the reported result.

Trials with low risk of bias were considered those trials having “low risk of bias” in all of the above specified individual domains. Trials with high risk of bias were considered those trials that were judged as trials having “uncertain risk of bias” or “high risk of bias” in one or more of the above specified individual domains. Disagreements were resolved by repeating the RoB assessment or by discussion with a third review author (A.B.).

### 2.6. Statistical Analysis

Dichotomous data were analyzed calculating the odds ratio (OR) for each trial, expressing the uncertainty with 95% confidence intervals (CI). Comparisons were made between trials evaluating oral and intravenous against intravenous-only antibiotic prophylaxis prior to elective laparoscopic surgery. The statistical package Review Manager 5.4 provided by The Cochrane Collaboration was used [31]. There were not any continuous variables in this review. The homogeneity among the included studies was assessed using the *I*^2^ statistic and the chi-squared test. An *I*^2^ value greater than 50% indicated substantial heterogeneity [29]. The fixed effects model was performed for studies with low heterogeneity (*I*^2^ ≤ 50%). In the presence of significant heterogeneity (*I*^2^ > 50%), the random effect model was used to pool the data. The pooled results were expressed by forest plots.

### 2.7. Certainty Assessment

Certainty (quality of evidence) of the body of evidence was assessed for each outcome of interest using the Grading of Recommendations, Assessment, Development and Evaluation (GRADE) approach and creating a Summary of Findings (SoF) table using the GRADEpro Guideline Development tool (GRADEpro GDT) [32]. The quality of evidence was classified according to the GRADE handbook [33] in one of four grades: (1) high, (2) moderate (3), low, or (4) very low. The quality of evidence could be downgraded by one (serious concern) or two levels (very serious concern) for the following reasons: risk of bias, inconsistency, indirectness, and imprecision.

## 3. Results

### 3.1. Study Selection

**Figure 1:**
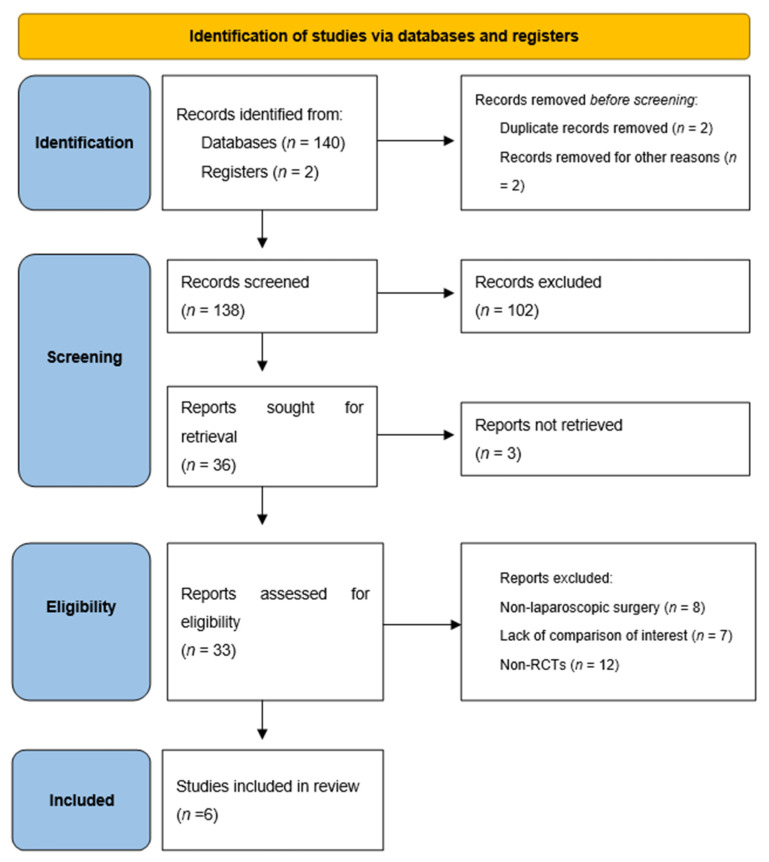
PRISMA 2020 flow diagram of the study.

A total of 137 studies were retrieved by the primary search. After deleting duplicates, records were screened. Following title and abstract screen, 102 clearly irrelevant records were excluded, leaving 35 potentially eligible studies of which full texts were retrieved. Twenty-nine studies were excluded because they did not meet the set criteria. Finally, 6 studies were eligible for the meta-analysis [34,35,36,37,38,39]. Figure 1 shows the detailed process of the studies selection.

The studies randomized a total of 2252 patients, of whom 1126 were in the oral + intravenous (Oral + IV) group and 1126 were in the intravenous-only (IV) group. All trials reported data about SSI, which was defined according to the guidelines issued by the Centers for Disease Control and Prevention [7] and classified as being either incisional or organ/space infection. Incisional SSIs were infections occurring at the incisional site within 30 days after the procedure and involving the skin, subcutaneous tissue, muscle and fascia but not the organ/space. The organ/space SSIs involved any part of the anatomy other than the incised body wall layers that had been opened or manipulated during the surgery [34,35].

Study characteristics, baseline data of patients, types of interventions, outcomes, and the details of antibiotic regimes administered are listed in Table 1.

### 3.2. Risk of Bias Assessment

One trial was at low risk of bias [34]. Two trials [35,37] showed some concerns, whereas three trials [36,38,39] were at high risk of bias according to RoB 2 [30]. The risk of bias assessment is reported in Appendix A Table A1.

### 3.3. Surgical Site Infections

**Figure 2:**
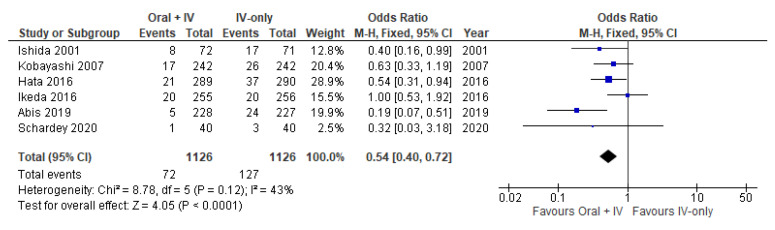
Forest plot of Overall SSIs comparing oral + IV group and IV-only group.

All six studies involving 2252 patients reported available data on the overall SSIs and low heterogeneity was observed among the trials (*I*^2^ = 43%). Therefore, the fixed effect model was used to pool the data. SSI occurred in 72 of 1126 patients in the intervention group (6.4%) vs. 127 of 1126 patients in the control group (11.3%). Meta-analysis results showed a statistically significant reduction of SSIs in the group of patients receiving Oral antibiotics in addition to IV antibiotics (OR 0.54, 95% CI 0.40 to 0.72; Figure 2).

**Figure 3:**
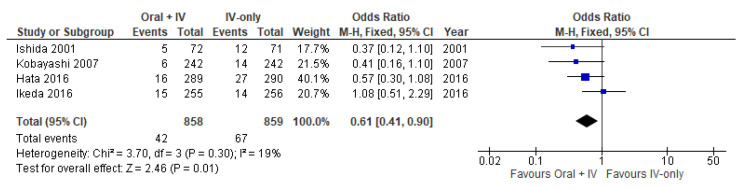
Forest plot of incisional SSIs comparing oral + IV group and IV-only group.

**Figure 4:**
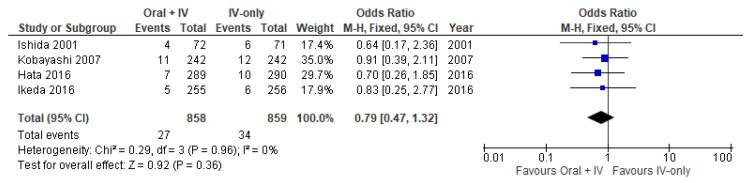
Forest plot of organ/space SSIs comparing oral + IV group and IV-only group.

Furthermore, four trials [34,35,36,39] involving 1717 patients reported data specifically on incisional SSIs and organ/space SSIs. Incisional SSI occurred in 42 of 858 patients in the intervention group (4.9%) vs. 67 of 859 patients in the control group (7.8%). Organ/space SSIs occurred in 27 of 858 patients in the intervention group (3.1%) vs. 34 of 859 patients in the control group (4%). Meta-analysis results showed a statistically significant reduction in incisional SSIs (OR 0.61, 95% CI 0.41 to 0.90; *I*^2^ = 19%) and a nonstatistically significant reduction organ/space SSIs (OR 0.79, 95% CI 0.47 to 1.32; *I*^2^ = 0%) in the group of patients receiving oral antibiotics in addition to IV antibiotics (Figure 3 and Figure 4).

### 3.4. Anastomotic Leakage

**Figure 5:**
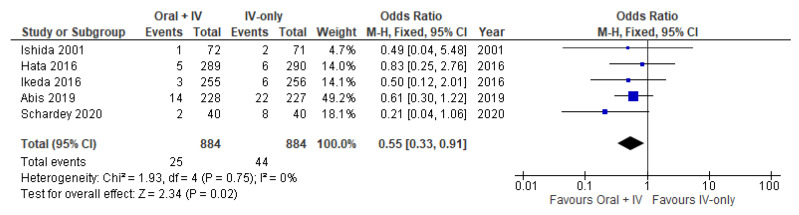
Forest plot of anastomotic leakage comparing oral + IV group and IV-only group.

Five studies [34,35,36,37,38] involving 1768 patients reported available data on anastomotic leak and no heterogeneity was observed among the trials (*I*^2^ = 0%). Therefore, the fixed effects model was performed to pool the data. Anastomotic leakage occurred in 25 out of 884 patients in the intervention group (2.8%) vs. 44 out of 884 patients in the control group (4.9%). Meta-analysis of the included studies showed a statistically significant protective effect in the group of patients receiving oral antibiotics in addition to IV antibiotics vs. IV antibiotic prophylaxis alone (OR 0.55, 95% CI 0.33 to 0.91; Figure 5).

### 3.5. Enteritis/Colitis

**Figure 6:**
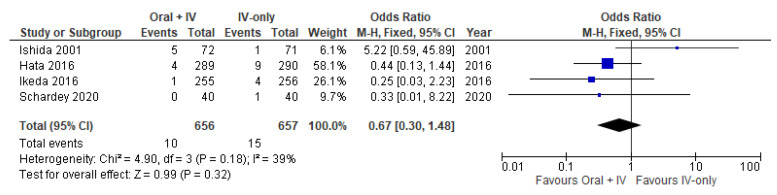
Forest plot of enteritis/colitis comparing oral + IV group and IV-only group.

Four studies [34,35,36,38] involving 1313 patients reported available data on enteritis/colitis. Low heterogeneity was observed among the trials (*I*^2^ = 39%). Therefore, the fixed effects model was performed to pool the data. Meta-analysis of the included studies showed no significant difference between the oral + IV and IV-only groups (OR 0.67, 95% CI 0.30 to 1.48; Figure 6).

### 3.6. Pneumonia

**Figure 7:**
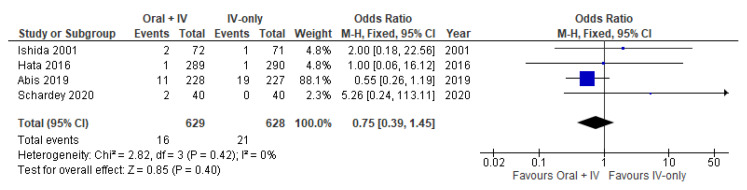
Forest plot of pneumonia comparing oral + IV group and IV-only group.

Four studies [35,36,37,38] involving 1257 patients reported available data on pneumonia and no heterogeneity was observed among the trials (*I*^2^ = 0%). Therefore, the fixed effects model was performed to pool the data. Meta-analysis of the included studies showed no significant difference between the oral + IV and IV-only groups (OR 0.75, 95% CI 0.39 to 1.45; Figure 7).

### 3.7. Urinary Tract Disorder

**Figure 8:**
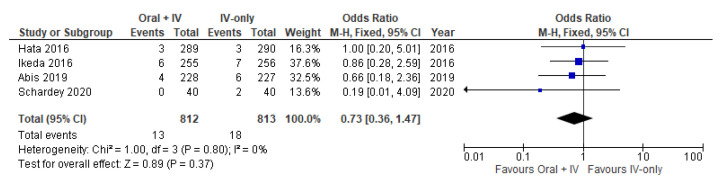
Forest plot of urinary tract disorder comparing oral + IV group and IV-only group.

Four studies [34,35,37,38] involving 1625 patients reported available data on urinary tract disorder and no heterogeneity was observed among the trials (*I*^2^ = 0%). Therefore, the fixed effects model was performed to pool the data. Meta-analysis of the included studies showed no significant difference between the oral + IV and IV-only group (OR 0.73, 95% CI 0.36 to 1.47; Figure 8).

### 3.8. Bowel Obstruction

**Figure 9:**
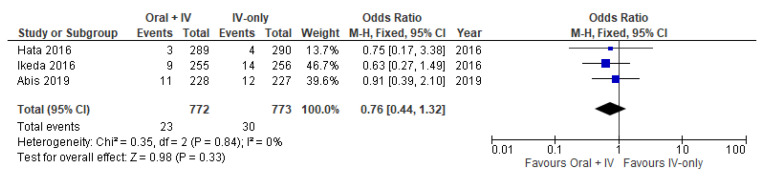
Forest plot of bowel obstruction comparing oral + IV group and IV-only group.

Three studies [34,35,37] involving 1545 patients reported available data on bowel obstruction and no heterogeneity was observed among the trails (*I*^2^ = 0%). Therefore, the fixed effects model was performed to pool the data. Meta-analysis of the included studies showed no significant difference between the oral + IV and IV-only groups (OR 0.76, 95% CI 0.44 to 1.32; Figure 9).

### 3.9. Certainty Assessment

Certainty (quality of evidence) in the body of evidence was assessed for both primary and secondary outcomes. According to the GRADE Handbook [33], certainty was moderate for all the outcomes except for bowel obstruction. The certainty assessment is detailed in SoF table created with GRADEpro GDT [32] (Table 2).

## 4. Discussion

Our meta-analysis showed that a combination of oral antibiotics and intravenous antibiotics could significantly lower the incidence of SSI compared with intravenous antibiotics alone in patients undergoing laparoscopic colorectal resection. Several studies concerning the administration route for antibiotics and the number of administrations showed a decrease in surgical site infection after colorectal cancer resection when antibiotic prophylaxis was applied [5,6,7,26,40,41,42]. Bellows and colleagues conducted a large meta-analysis of RCTs to compare the effectiveness of combined oral nonabsorbable and intravenous antibiotics to reduce SSIs [26]. The results of this study found that patients receiving a combination of oral and IV antibiotics had decreased SSIs compared to patients receiving only IV antibiotics. Thus, the authors concluded that both oral nonabsorbable and IV antibiotics are necessary. However, this analysis included studies that combined laparoscopic and open surgical procedures. The protocol of antibiotic regiments was not standardized. We have also analyzed whether adding oral antibiotics to intravenous antibiotics had any effect on anastomotic leakage. Our results indicated that patients randomly assigned to an oral antibiotic regimen in addition to intravenous antibiotics had a statistically significant reduced risk of anastomotic leakage compared with participants receiving only intravenous antibiotics. Reduced rates of AL in patients receiving oral antibiotic prophylaxis were shown in earlier studies [43,44,45]; however, indications for surgery varied considerably in these studies and did not only include CRC, but also inflammatory bowel disease (IBD), fistulas and other benign and/or infectious diseases that have a different postoperative complication risk. Furthermore, patients treated for IBD often used immunosuppressive medication, which would influence the incidence on infectious complications and tissue healing. The present meta-analysis comprised of a more homogenous patient group, focusing only on patients undergoing elective laparoscopic resection for colorectal cancer. Furthermore, all the included studies of our meta-analysis were published after 2000, this can ensure the quality of our results because the implementation of enhanced recovery after surgery (ERAS) protocols and laparoscopic surgery in this period have shown to reduce the incidence of postoperative infections [46]. Moreover, most of the previous systematic reviews and meta-analysis mostly focused upon SSI alone, whereas this analysis determined the effect of oral antibiotic prophylaxis on AL as well.

Regarding other infectious and noninfectious postoperative complications (enteritis/colitis, pneumonia, urinary tract disorder, and bowel obstruction), the magnitudes of the pooled effects showed a protective trend, although not statistically significant. The explanation of these results may be due to the fact that only few of the included studies reported data about the mentioned outcomes, therefore we may lack of power to detect true effects. Other explanations could have make these results less generalizable to other patient populations. Iatrogenic ureteral injuries are often reported as a result of laparoscopic surgery [47]; this constitutes an important risk factor associated with postoperative urinary retention that increases the risk of developing urinary tract infection, particularly for geriatric females undergoing the rectal procedure with preoperative steroid use, prolonged duration of the surgery, and under higher classes of anesthesia [48]. A study has shown that laparoscopic and open surgery are equally associated with the development of bowel obstruction [49], and this may be due to several risks factor, such as male sex, emergency surgery, longer duration of surgery, and dysfunctional ileostomy placement [50].

The present study has several limitations. First, there is a wide variation in the type, timing, and dosing of oral antibiotics regimes, both selective [37] and broad-spectrum oral antibiotic prophylaxis have been reported in the included studies. Selective antibiotic prophylaxis is known to target only specific bacteria while leaving indigenous anaerobic, bacteria largely undisturbed [51]. The disadvantage of broad-spectrum oral antibiotic therapy is that it results in a more extensive elimination of bacteria possibly leading to microbial dysbiosis. Therefore, the data supporting the use of universal preoperative oral antibiotic regimens and route of administration for colorectal surgery remains unclear. Second, the use of enhanced recovery protocols is not always documented, which are also known to impact patient outcomes. Third, we did not discriminate between nonabsorbable and absorbable antibiotics given by the oral route. Another important point to discuss is outcome reporting bias. In our study, all of the trials analyzed included data about SSI. However, a certain variability in reporting the secondary outcomes was detected, which supports the moderate certainty of evidence presented in our SoF table and explains the low certainty assessed for the outcome bowel obstruction. Furthermore, it must be highlighted that the three trials [36,38,39] reported as high risk of bias shared concerns in measurement of the outcome and in selection of the reported result, which supports even more these grades of evidence. In many studies, a range of outcome measures is recorded but not all are reported [52]. The choice of outcomes that are reported can be influenced by the results, potentially making published results misleading [53]. Despite these limitations, our study has several key strengths. The main strength of this systematic review and meta-analysis is that, to the best of our knowledge, it is the first study evaluating the role of oral antibiotic prophylaxis in a homogeneous group of patients undergoing elective laparoscopic CRC surgery in a contemporary setting. Even with this focused review, the size of our study was still substantial. We also assessed the risk of bias according to Cochrane Handbook and used GRADE approach to determine the quality of evidence. The methodological quality of included RCTs was not poor. Moreover, the quality of evidence of almost each finding according to the GRADE approach was moderate. Therefore, we can conclude that the use of preoperative oral antibiotics in laparoscopic colorectal surgery should be encouraged.

In conclusion, our results showed that adding an oral antibiotic prophylaxis could have a positive impact in reducing the incidence of SSI after laparoscopic colorectal surgery. However, previous studies have showed that skin preparation, the timing and method of wound closure, patients’ comorbidity, intraoperative body temperature, and anatomic location of the colonic resection are significant factors that can also influence the incidence of subsequent infection [54,55,56]. Furthermore, RCTs assessing all the variables that can adversely affect the incidence of infectious complications should be carried out in order to establish the best strategy to decrease SSI after laparoscopic colorectal surgery.

## Figures and Tables

**Table 1:** Study characteristics of included studies.

Study	Design	Total *N*	Intervention (Oral + IV) *N*	Control (IV-Only) *N*	Primary Outcome	Secondary Outcome	Oral Antibiotics	IV Antibiotics	Type of Resection
Ishida 2001 [36]	RCT	143	72	71	SSI	Anastomotic leak, Enteritis/colitis, Pneumonia	Kanamycin 500 mg + Erythromycin 400 mg in 4 daily doses, started 2 days preoperatively + control group treatment	Cefotiam 1 g in 2 daily doses for 48 h	Colectomy—76 Anterior resection—47 APR—9 Total proctectomy with J pouch—3 Total pelvic exenteration—4 Other—4
Kobayashi 2007 [39]	RCT	484	242	242	SSI	/	Kanamycin 1 g + Erythromycin 400 mg at 14:00, 15:00, and 23:00 + control group treatment	Cefmetazole 1 g after the induction of anesthesia, additional dose if the operation was prolonged beyond 3 h. Again twice daily for 3	Surgical procedure: Colon—241 Rectum—243
Ikeda 2016 [34]	RCT	511	255	256	SSI	Anastomotic leak, Enteritis/colitis, Urinary tract disorder, Bowel obstruction	Kanamycin 1000 mg 2 doses + Metronidazole 750 mg, started 1 day preoperative + control group treatment	Cefmetazole 1 g 3 doses in 24 h	Colonic surgery—309 Anterior resection—177 APR—25
Hata 2016 [35]	RCT	579	289	290	SSI	Anastomotic leak, Enteritis/colitis; Pneumonia, Urinary tract disorder, Bowel obstruction	Kanamycin 1 g + Metronidazole 750 mg at 13 h and 9 h before the surgery + control group treatment	Cefmetazole 1 g was administered intravenously 30 min before the skin incision, additional dose was given every 3 h during the surgery	Colectomy—376 Anterior resection—183 APR—20
Abis 2019 [37]	RCT	455	228	227	SSI	Anastomotic leak, Pneumonia, Urinary tract disorder, Bowel obstruction	SDD 3 days prior to surgery until 3 days after surgery or when normal bowel motion occurred + control group treatment	Cefazoline 1 g + Metronidazole 500 mg, intravenously, 30 min prior to skin incision	Right hemicolectomy—162 Transverse colectomy—17 Left hemicolectomy—41 Sigmoid resection—124 Low anterior resection—103 Other—8
Schardey 2020 [38]	RCT	80	40	40	SSI	Anastomotic leak, Pneumonia, Enteritis/colitis, Urinary tract disorder	Polymyxin B sulphate 100 mg + Tobramycin 80 mg + Vancomycin 125 mg + Amphotericin B 500 mg 4 daily doses, started 1 day preoperatively until day 7 postoperatively.	Amphotericin B 500 mg + Lactulose 305 mg	Low anterior resection with TME—80

**Table 2:** Summary of Findings (SoF) table.

Oral and Parenteral vs. Parenteral Antibiotic Prophylaxis for Patients Undergoing Laparoscopic Colorectal Resection					
**Patient or population:** Patients undergoing laparoscopic colorectal resection **Setting:** Multicentered study **Intervention:** Oral and parenteral antibiotic prophylaxis **Comparison:** Parenteral antibiotic prophylaxis only					
**Outcomes**	**№ of Participants (Studies)** **Follow-Up**	**Certainty of the Evidence (GRADE)**	**Relative** **Effect** **(95% CI)**	**Anticipated Absolute Effects**	
				**Risk with Parenteral Antibiotic Prophylaxis**	**Risk Difference with Oral and Parenteral Antibiotic Prophylaxis**
Overall Surgical Site Infections	2252 (6 RCTs)	⨁⨁⨁◯ Moderate	OR 0.54 (0.40 to 0.72)	113 per 1000	49 fewer per 1000 (64 fewer to 29 fewer)
Incisional Surgical Site Infections	1717 (4 RCTs)	⨁⨁⨁◯ Moderate	OR 0.61 (0.41 to 0.90)	78 per 1000	29 fewer per 1000 (44 fewer to 7 fewer)
Organ/space Surgical Site Infections	1717 (4 RCTs)	⨁⨁⨁◯ Moderate	OR 0.79 (0.47 to 1.32)	40 per 1000	8 fewer per 1000 21 fewer to 12 more)
Anastomotic Leakage	1768 (5 RCTs)	⨁⨁⨁◯ Moderate	OR 0.55 (0.33 to 0.91)	50 per 1000	22 fewer per 1000 (33 fewer to 4 fewer)
Enteritis/colitis	1313 (4 RCTs)	⨁⨁⨁◯ Moderate	OR 0.67 (0.30 to 1.48)	23 per 1000	7 fewer per 1000 (16 fewer to 11 more)
Pneumonia	1257 (4 RCTs)	⨁⨁⨁◯ Moderate	OR 0.75 (0.39 to 1.45)	33 per 1000	8 fewer per 1000 (20 fewer to 14 more)
Urinary Tract Disorder	1625 (4 RCTs)	⨁⨁⨁◯ Moderate	OR 0.73 (0.36 to 1.47)	22 per 1000	6 fewer per 1000 (14 fewer to 10 more)
Bowel Obstruction	1545 (3 RCTs)	⨁⨁◯ ◯ Low	OR 0.76 (0.44 to 1.32)	39 per 1000	9 fewer per 1000 (21 fewer to 12 more)
**The risk in the intervention group** (and its 95% confidence interval) is based on the assumed risk in the comparison group and the **relative effect** of the intervention (and its 95% CI). **CI:** confidence interval; **OR:** odds ratio					
**GRADE Working Group grades of evidence** **High certainty:** We are very confident that the true effect lies close to that of the estimate of the effect. **Moderate certainty:** We are moderately confident in the effect estimate: the true effect is likely to be close to the estimate of the effect, but there is a possibility that it is substantially different. **Low certainty:** Our confidence in the effect estimate is limited: the true effect may be substantially different from the estimate of the effect. **Very low certainty:** We have very little confidence in the effect estimate: the true effect is likely to be substantially different from the estimate of effect.					

## Abbreviations

SSIs: Surgical Site Infections; AL, Anastomotic leakage; RCTs, randomised clinical trials; RoB 2, Cochrane revised tool for assessing risk of bias in randomised trials; PAP, Perioperative antibiotic prophylaxis; PRISMA, Preferred Reporting Items for Systematic reviews and Meta-Analyses; PRESS, Peer Review of Electronic Search Strategies; CENTRAL, The Cochrane Central Register of Controlled Trials; CD, Clostridium Difficile; OR, odds ratio; CI, confidence intervals; GRADE, Grading of Recommendations, Assessment, Development and Evaluation; GRADEpro GDT, GRADEpro Guideline Development tool; IV, intravenous.

## Appendix A. Search Strategy

**Table:**

Database	Date of Search	Search Strategy
Cochrane Central Register of Controlled Trials (Central)	1 May 2021, updated 6 December 2021	#1 MeSH descriptor: [Laparoscopy] explode all trees #2 MeSH descriptor: [Colorectal Neoplasms] explode all trees #3 MeSH descriptor: [Antibiotic Prophylaxis] explode all trees #4 MeSH descriptor: [Surgical Wound Infection] explode all trees #5 Laparoscop * AND Colorectal neoplasm* #6 antibiotic * OR prophyla* #7 #1 AND #2 #8 #5 OR #7 #9 #3 OR #6 #10 surgical wound * OR wound * infection * #11 #4 OR #10 #12 #8 AND #9 #11
PUBMED (Ovid SP)	1 May 2021, updated 6 December 2021	(1) “laparoscopie”[All Fields] OR “laparoscopy”[MeSH Terms] OR “laparoscopy”[All Fields] OR “laparoscopies”[All Fields] (2) “colorectal neoplasms”[MeSH Terms] OR (“colorectal”[All Fields] AND “neoplasms”[All Fields]) OR “colorectal neoplasms”[All Fields] OR (“colorectal”[All Fields] AND “neoplasm”[All Fields]) OR “colorectal neoplasm”[All Fields] (3) “antibiotic prophylaxis”[MeSH Terms] OR (“antibiotic”[All Fields] AND “prophylaxis”[All Fields]) OR “antibiotic prophylaxis”[All Fields] (4) “oral”[All Fields] OR “mouth”[MeSH Terms] OR “mouth”[All Fields] (5) “infusions, parenteral”[MeSH Terms] OR (“infusions”[All Fields] AND “parenteral”[All Fields]) OR “parenteral infusions”[All Fields] OR (“infusions”[All Fields] AND “parenteral”[All Fields]) OR “infusions, parenteral”[All Fields] (6) “surgical wound infection”[MeSH Terms] OR (“surgical”[All Fields] AND “wound”[All Fields] AND “infection”[All Fields]) OR “surgical wound infection”[All Fields] (7) 1 AND 2 (8) 3 OR 4 OR 5 (9) 7 AND 8 AND 6
EMBASE (Ovid SP)	1 May 2021, updated 6 December 2021	1. “laparoscopie”[All Fields] OR “laparoscopy”[MeSH Terms] OR “laparoscopy”[All Fields] OR “laparoscopies”[All Fields] 2. “colorectal neoplasms”[MeSH Terms] OR (“colorectal”[All Fields] AND “neoplasms”[All Fields]) OR “colorectal neoplasms”[All Fields] OR (“colorectal”[All Fields] AND “neoplasm”[All Fields]) OR “colorectal neoplasm”[All Fields] 3. “antibiotic prophylaxis”[MeSH Terms] OR (“antibiotic”[All Fields] AND “prophylaxis”[All Fields]) OR “antibiotic prophylaxis”[All Fields] 4. “oral”[All Fields] OR “mouth”[MeSH Terms] OR “mouth”[All Fields] 5. “infusions, parenteral”[MeSH Terms] OR (“infusions”[All Fields] AND “parenteral”[All Fields]) OR “parenteral infusions”[All Fields] OR (“infusions”[All Fields] AND “parenteral”[All Fields]) OR “infusions, parenteral”[All Fields] 6. “surgical wound infection”[MeSH Terms] OR (“surgical”[All Fields] AND “wound”[All Fields] AND “infection”[All Fields]) OR “surgical wound infection”[All Fields] 7. 1 AND 2 8. 3 OR 4 OR 5 9. 7 AND 8 AND 6
Science Citation Index Expanded	6 December 2021	# 5 #4 AND #3 # 4 TS=(surgical wound infection *) # 3 #2 AND #1 # 2 TS=(laparoscop * and colorectal neoplasm *) # 1 TS=(antibiotic * or prophyla * or oral or parenteral)

* indicates the retrieval of all forms of the word.

**Table A1:** Risk of bias assessment of included studies.

**Ishida 2001**	
**Risk of bias arising from the randomization process**	Some concerns
Risk of bias due to deviations from the intended interventions	Low
Bias due to missing outcome data	Low
Risk of bias in measurement of the outcome	High
Risk of bias in selection of the reported result	Some concerns
**Overall risk of bias**	**High**
**Kobayashi 2007**	
**Risk of bias arising from the randomization process**	Low
Risk of bias due to deviations from the intended interventions	Some concerns
Bias due to missing outcome data	Low
Risk of bias in measurement of the outcome	High
Risk of bias in selection of the reported result	High
**Overall risk of bias**	**High**
**Ikeda 2016**	
**Risk of bias arising from the randomization process**	Low
Risk of bias due to deviations from the intended interventions	Low
Bias due to missing outcome data	Low
Risk of bias in measurement of the outcome	Low
Risk of bias in selection of the reported result	Low
**Overall risk of bias**	**Low**
**Hata 2016**	
**Risk of bias arising from the randomization process**	Low
Risk of bias due to deviations from the intended interventions	Some concerns
Bias due to missing outcome data	Low
Risk of bias in measurement of the outcome	Low
Risk of bias in selection of the reported result	Low
**Overall risk of bias**	**Some concerns**
**Abis 2019**	
**Risk of bias arising from the randomization process**	Low
Risk of bias due to deviations from the intended interventions	Some concerns
Bias due to missing outcome data	Low
Risk of bias in measurement of the outcome	Low
Risk of bias in selection of the reported result	Low
**Overall risk of bias**	**Some concerns**
**Schardey 2020**	
**Risk of bias arising from the randomization process**	Low
Risk of bias due to deviations from the intended interventions	Low
Bias due to missing outcome data	Low
Risk of bias in measurement of the outcome	Some concerns
Risk of bias in selection of the reported result	High
**Overall risk of bias**	**High**
